# Prevalence of Peri-Implant Mucositis, Peri-Implantitis and Associated Risk Indicators of Implants with and without Laser-Microgrooved Collar Surface: A Long-Term (≥20 Years) Retrospective Study

**DOI:** 10.3390/jpm14040342

**Published:** 2024-03-25

**Authors:** Renzo Guarnieri, Rodolfo Reda, Dario Di Nardo, Francesco Pagnoni, Alessio Zanza, Luca Testarelli

**Affiliations:** 1 Private Periodontal Implant Practice, 31100 Treviso, Italy; renzoguarnieri@gmail.com; 2 Department of Oral and Maxillo-Facial Sciences, Sapienza University of Rome, 00161 Rome, Italy; francesco.pagnoni22@gmail.com (F.P.); alessio.zanza@uniroma1.it (A.Z.); luca.testarelli@uniroma1.it (L.T.); 3 Dentistry Department, Fondazione Policlinico Universitario Campus Bio-Medico di Roma, Via Álvaro del Portillo 5, 00128 Rome, Italy; d.dinardo@policlinicocampus.it

**Keywords:** implants, laser-microgrooved collar surface, implant neck, peri-implantitis, peri-implant mucositis

## Abstract

The aim of the current study was to retrospectively investigate the prevalence of peri-implant mucositis (PIM) and peri-implantitis (P) in a long-term follow-up (≥20 years) of implants with the same body design and body surface but different collar surfaces with laser-microtextured grooves (LMGSs) vs. no laser-microtextured grooves (no-LMGSs) in private practice patients. Furthermore, several patient-related, implant-related, site-, surgical-, and prosthesis-related potential disease risk factors were analyzed. A chart review of patients receiving at least one pair of implants (one with an LMGS and the other without LMGS) in the period 1993–2002 was used. Chi-square analysis was used to determine if a statistically significant difference between the investigated variables and PIM/P was present. Possible risk factors were statistically evaluated by a binary logistic regression analysis. A total of 362 patients with 901 implant-supported restorations (438 with LMGS and 463 no-LMGS) were included in the study. The cumulative survival rates of implants at 5, 10, 15, and 20 years were 98.1%, 97.4%, 95.4%, and 89.8%, respectively, for the LMGS group, and 93.2%, 91.6%, 89.5%, and 78.3% for the no-LMGS group. The difference was statistically significant at all timepoints (*p* < 0.05). In total, at the end of the follow-up period, 45.7% of patients and 39.8% of implants presented PIM, and 15.6% of patients and 14% of implants presented P. A total of 164 LMGS implants (37.4%) and 195 no-LMGS implants (42.1%) presented peri-implant mucositis, while 28 (6.3%) of LMGS implants and 98 (21.1%) no-LMGS implants demonstrated peri-implantitis. Differences between LMGS implants and no-LMGS implants were statistically significant (*p* < 0.05). The binary logistic regression identified collar surface, cigarette smoking, histories of treated periodontitis, and lack of peri-implant maintenance as risk factors for P. After at least 20 years of function in patients followed privately, LMGS implants compared to no-LMGS implants presented a statistically and significantly lower incidence of P. Implant collar surface, cigarette smoking, previously treated periodontitis, and lack of peri-implant maintenance are factors with significant association to P.

## 1. Introduction

Although prosthetic oral rehabilitation using dental implants is considered a safe and predictable therapy in partially and totally edentulous patients [1,2], biological complications can reduce success and survival rates over the long term [3,4,5]. The most recent consensus conference, which took place in 2017, has classified implant biological complications as either peri-implant mucositis (PIM) or peri-implantitis (P) [6]. In PIM the inflammation affects only the soft tissues [7], while in P it involves the supporting bone, which presents a progressive loss beyond physiological bone remodeling [8]. It is believed that both have a microbiological etiology [6] and that P represents the aggravating evolutionary stage of PIM [6]. However, regarding the role of bacteria in the development of P, many questions remain unclear: microbiological implant contamination appears to be necessary but not sufficient, as it is the immunological response of the host that supports the pathophysiology of P, with the continuous and progressive involvement of the peri-implant support bone [6,7,8].

Many studies and systematic reviews have reported the prevalence of PIM and P, with a great variability of results [9,10,11,12,13]. Prevalence of PIM ranged between 9.7% and 64.6%. Since the diagnosis of PIM is based only on the presence of bleeding on probing (BoP), which is affected by biofilm accumulation, PIM prevalence generally depends on the type of population included in studies [7,8]. In fact, the prevalence of PIM tends to be low when patients maintain good oral hygiene control and are enrolled in supportive maintenance therapy [7]. The prevalence of P has been reported to range from 4.7% to 45% at the patient level and from 3.6% to 22.1% at the implant level. The variability in the prevalence of P, reported by various studies, depends on the different clinical and radiological thresholds of probing depth and bone loss used to diagnose the disease [6,13]. Several factors have been identified as being at risk of the onset of PIM and P [14]. In this case, a recent review [6] indicated that poor plaque control, absence of periodontal maintenance, presence of previously treated periodontitis, inadequate implant positioning, suprastructures with over contours, presence of cement overflows, and lack of keratinized mucosa are strongly associated with the development of P. All these factors reduce the subject’s ability to remove bacterial plaque. Other factors, such as genetics, smoking habits, alcohol habits, diabetes, osteoporosis, occlusal overload, implant and collar surface characteristics, and implant collar/platform designs, have been reported with conflicting evidence [14]. Different implant collar surfaces (smooth, rough, micro-threaded, or laser micro-grooved) and implant designs (straight, scalloped, butt joint, or platform switched) have been proposed over the years to improve the “performance” of dental implants. A laser micro-grooved surface was developed to influence the way different cell lineages could interact with the titanium surface. Pre-clinical studies [15,16], evaluating the impact of surface microgeometry on the in vitro behavior of fibroblasts, showed a parallel orientation and a channeling of these cells on microgrooved surfaces, whereas fibroblasts grown on non-grooved surfaces showed random orientation. Furthermore, oriented cell filipodial contacts with fibrin fibril in a parallel orientation were present in laser-ablated microgrooves [17]. These in vitro outcomes allowed us to hypothesize that laser-microgrooved surfaces could be used to influence soft tissue responses to collar implant surfaces. Subsequent histological studies in humans have validated this hypothesis, documenting the presence of a perpendicular connective fiber orientation with a physical attachment onto laser-produced microgrooves on implant collars [18]. Since physical, chemical, and micro-geometric implant features of the collar surface, exposed to the oral cavity, could influence plaque retention and, consequently, predisposition to PIM and P, they have long been a subject for discussion. However, little data have been published on what influence the implant collar surface microgeometry can have on the onset and development of peri-implant biological complications [19,20,21].

The aim of the current study was to retrospectively evaluate the prevalence of biological complications, such as PIM and P, and risk indicators as observed in private practice patients receiving at least one pair of implants with the same body design and surface, but with different collar surfaces [one with a laser-microtextured groove (LMGS) and the other without LMGS] in the long-term follow-up (>20 years).

## 2. Materials and Methods

### 2.1. Study Design and Participants

The current retrospective study included adult patients receiving at least one LMGS implant and one no-LMGS dental implant placed in private practice in Italy during the period from 1993 to 2002. Clinical and radiographic evaluation of all patients was performed prior to surgery and during the follow-up visits up until 2023. All patients were invited to adhere to a six-month supportive implant therapy. Age, sex, systemic and dental health data, and frequency of recall visits were recorded for each patient. The STROBE checklist was used to design and conduct the study [22]. 

Inclusion Criteria: aged ≥18 years; patients received at least one LMGS- and one no-LMGS-implant placed in the period from 1993 to 2002. 

### 2.2. Exclusion Criteria

Records with missing initial and final periodontal parameters, radiographic measurements, and data on more than 50% of the follow-up time were excluded. Subjects with unstable periodontal health status during the study’s period and with insufficient clinical and radiographic data at each recall visit were also excluded.

### 2.3. Implants

**Figure 1:**
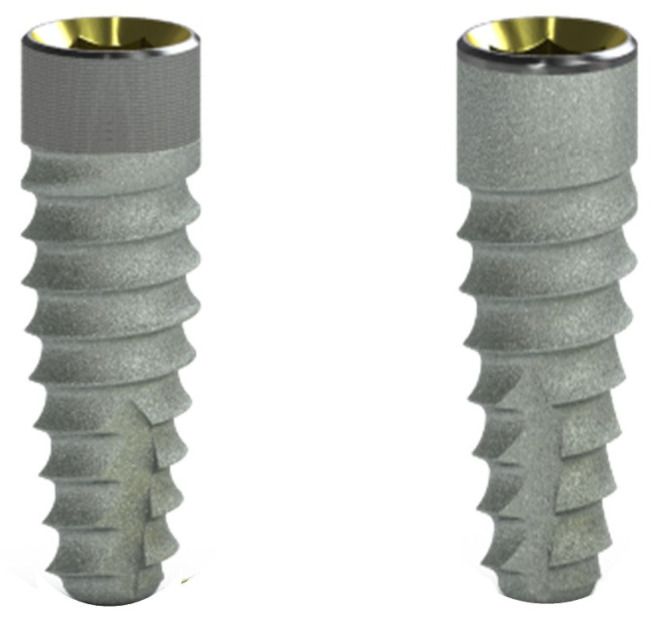
Implants investigated in the present study include implants with a laser-microgrooved collar surface (LMGS, **left**) and implants without a laser-microgrooved collar surface (no-LMGS, **right**).

Implants investigated in the current study (Tapered implants, BioHorizons, Birmingham, AL, USA) have the same body design and body surface but with different collar surfaces. The laser-microgrooved collar implant has 1.8 mm laser-produced microtextured grooves, while the implant without laser-microtextured grooves has the collar surface machined (0.3 mm) and grit-blasted (1.5 mm) (Figure 1). Part of the implants examined were randomly positioned in an alternating sequence. The selection of other implants was based on the patient’s or dentist’s choice, taking care to minimize variations and select sites that were as similar as possible in this regard. Previous publications of the authors reported a detailed description of the clinical, surgical, radiographic, and prosthetic procedures [23,24].

### 2.4. Ethical Approval

All patients selected for this study had already been treated on the basis of previously approved research protocols; therefore, approval from a new ethics committee was not necessary. Each patient was informed about the aims of this study and signed the informed consent drawn up on the basis of the Declaration of Helsinki.

### 2.5. Data Collection 

The following data were recorded by means of dedicated software (XDent CGM Italia Group 2021) by one of the clinicians (RG).

(1) Demographic data.
(2) Systemic/patient-related factors (gender, systemic conditions, diabetes mellitus, dyslipidemia, hypertension, osteoporosis, anemia, hypothyroidism, smoking habits, history of treated periodontitis, and lack of regular peri-implant maintenance (<1 dental maintenance visit per year).
(3) Implant/site/surgical-related factors (implant collar surface [LMGS vs. no-LMGS, location, height, diameter, placement protocol, number of functional years prior to PIM/P, use of grafting materials at the time of implant placement, and presence/absence of a wide band of keratinized mucosa (2 mm or more).
(4) Prosthesis-related factors (type of retention, number of functional years prior to diagnosis, screw loosening, crown chipping, and crown debonding).

The frequencies of maintenance visits were also collected and analyzed as possible risk factors for P.

### 2.6. Case Definition 

Healthy implant P-IM and P were diagnosed according to the definition proposed by the 2017 World Guidelines [6]. 

Healthy implant: no BoP, no bone loss beyond the limits of the initial physiological remodeling. 

PIM: the presence of BoP, no bone loss beyond the limits of the initial physiological remodeling. 

P: the presence of BoP and/or suppuration, bone loss beyond the limits of the initial physiological remodeling (>2 mm if absent initial parameters).

### 2.7. Power Analysis 

The 95% power and the 5% error, using G power software (version 3.1.9.2), were adopted for the sample size calculation. By including at least 15 risk factors, a representative sample size of 362 implants was needed. To account for possible exclusions, a total of 900 implants were included.

### 2.8. Statistical Analyses 

Statistical analyses were performed using the SAS software version 9.4. Chi-square analysis was used to determine if a statistically significant difference between the investigated variables and the prevalence of PIM/P was present. A *p* < 0.05 was considered statistically significant. Estimates of relative risk were also calculated for all variables. The patient was considered the unit of analysis for systemic and patient-related factors. To enhance the statistical accuracy, only one event of PIM or P per patient was included in the analysis. The implant was considered as the statistical unit for implant-, site-, and prosthesis-related factor analysis. Possible risk factors were dichotomized (P = 0 in the absence of events; P = 1 in the presence of events) and statistically evaluated by a binary logistic regression analysis. 

## 3. Results

A total of 362 patients with 901 implant-supported restorations (438 with LMGS and 463 without LMGS) were included in this study. The age of the patients ranged between 19 and 64 with a mean age of 41.9 ± 10.3 years. The cumulative survival rates for patients with LMGS implants at 5, 10, 15, and 20 years were 98.1, 97.4, 95.4, and 89.8%, respectively, and 93.2, 91.6, 89.5, and 78.3%, respectively, for no-LMGS implants. The difference was statistically significant at each timepoint. In total, at the end of the follow-up period, the prevalence of PIM at the patient and implant levels were 45.7% and 39.8%, respectively. For P, the prevalence at the patient level was 15.6%, while the prevalence at the implant level was 14.0%. 

**Figure 2:**
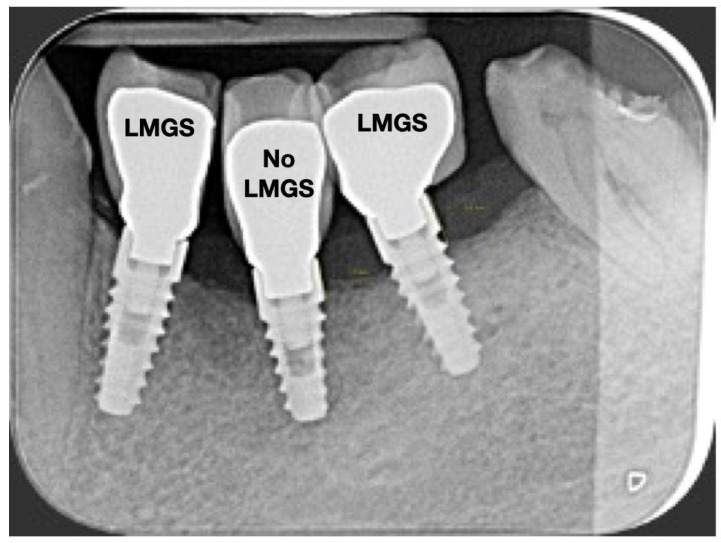
Radiograph of three (3) implants at crown delivery. (LMGS = with a laser-microgrooved collar surface; No-LMGS = without a laser-microgrooved collar surface).

**Figure 3:**
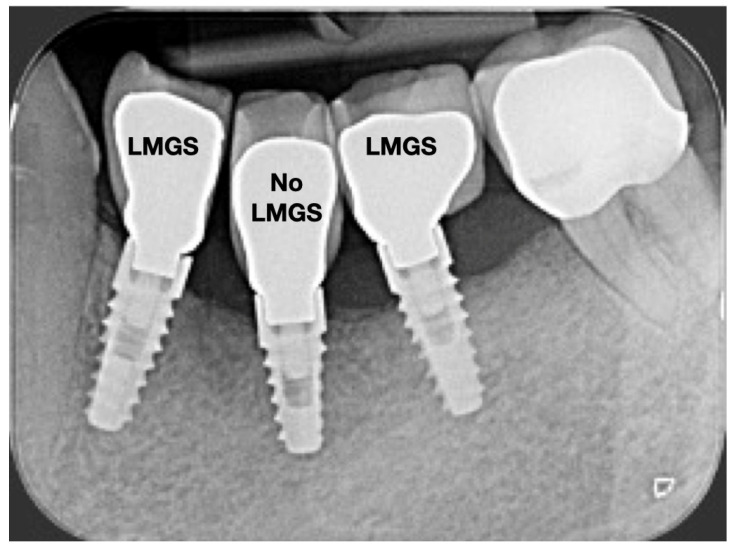
Radiograph of the same three (3) implants at 5 years. (LMGS = with a laser-microgrooved collar surface; No-LMGS = without a laser-microgrooved collar surface).

**Figure 4:**
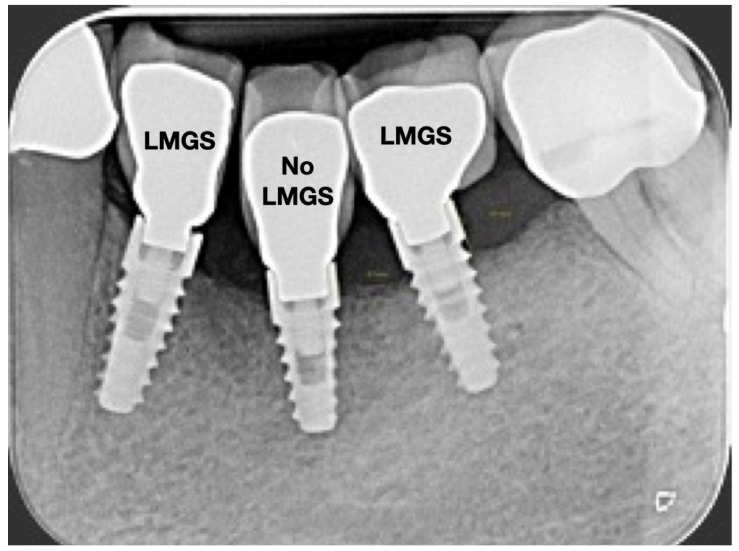
Radiograph of the same three (3) implants at 10 years. (LMGS = with a laser-microgrooved collar surface; No-LMGS = without a laser-microgrooved collar surface).

**Figure 5:**
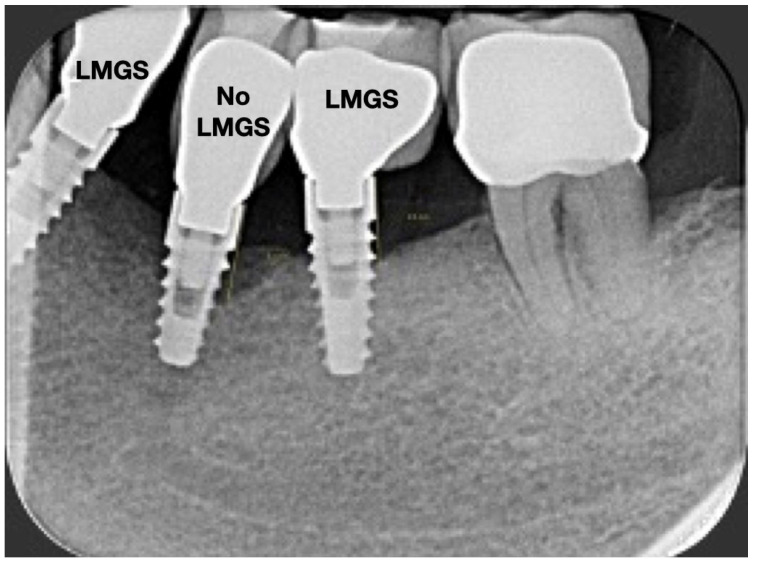
Radiograph of the same three (3) implants at the 15-year follow-up. Signs of P can be noted around the No-LMCS implant. (LMGS = with a laser-microgrooved collar surface; No-LMGS = without a laser-microgrooved collar surface).

A total of 164 LMGS implants (37.4%) and 195 no-LMGS implants (42.1%) presented PIM. Differences between LMGS implants and no-LMGS implants were not statistically significant (*p* > 0.05). A total of 28 (6.3%) LMGS implants and 98 (21.1%) no-LMGS implants demonstrated P with a statistically significant difference (*p* < 0.01). Figure 2, Figure 3, Figure 4 and Figure 5 report examples of radiographs during follow-up.

### 3.1. PIM

PIM was diagnosed more frequently in patients who irregularly attended maintenance visits (52.8% compared to 28.4% of regular attenders, [*p* < 0.01]). No significant association between PIM and other evaluated risk factors was noted (Table 1 and Table 2).

### 3.2. P

P was diagnosed more frequently in smokers, patients with previously treated periodontitis, and patients who did not attend regular peri-implant supportive therapeutic programs. No significant correlation between the prevalence of P, gender, and the presence of any systemic conditions was found (Table 3). 

The implant collar surface (no-LMCS) was found statistically correlated with P. Other implant-, site-, surgical-, and prosthesis-related factors showed no significant statistical correlation with the prevalence of P (Table 4).

A statistically significant association between P and smoking habits, previously treated periodontitis, lack of peri-implant supportive therapy, and no-LMCS was found using the binary logistic regression. The calculation of the odds ratio indicated that in the presence of these factors, the risk of peri-implantitis was, respectively, six, nine, seven, and eleven times higher (Table 5).

## 4. Discussion

The aim of the current retrospective study was to evaluate the prevalence of PIM and P and corresponding risk factors over a period of at least 20 years in private practice patients who received at least one implant with LMCS and one implant without LMCS. The use of two different implant collar surfaces in the same patient, according to the authors’ intentions, could have strengthened the significance of the results. 

The prevalence rates for PIM were 45.7% and 39.8% at patient and implant levels, respectively. Zitzmann and Berglundh [27] and Rinkee et al. [28] reported a PIM prevalence ranging from 39.4% to 80.0%, while Marrone et al. [29] and French et al. [30] observed a prevalence of 31.0% and 38.6%, respectively. In a systematic review, Deks and Tomasi [31] reported a PIM prevalence between 19% and 64.6%, with average values of 42.9% for the meta-analysis of studies. It is commonly accepted that PIM is an inflammatory reversible disease caused by the accumulation of dental biofilm, which affects the peri-implant mucosa without involving the supporting bone [7,8]. Since it is diagnosed only by the presence of BoP, which is affected by oral hygiene, the different prevalence reported by other studies could depend on the study population [7]. In the present study, 52.8% of patients diagnosed with PIM had a lack of regular supportive therapy, whereas only 28.4% of regular attendees were diagnosed with PIM. These outcomes are aligned with data recently reported by two literature reviews, indicating that the prevalence of PIM is lower in patients who adhere to regular supportive therapy [11,32]. In the present study, no statistically significant correlation was found between the type of implant collar surface and the prevalence of PIM. This supports previously reported outcomes, which underlined that the inflammatory response of the peri-implant soft tissue is not influenced by the type of surface implant collar/abutment surfaces but by the quantity and quality of the bacterial biofilm [19,20]. 

The prevalence rates for P recorded in the current study were 45.7% and 39.8% at patient and implant levels, respectively. The prevalence of P reported in the literature ranges from 4.7% to 45% at the patient level and from 3.6% to 22.1% at the implant level [9,10,11]. It is difficult to compare data recorded in the current study with other published data due to differences in study design and clinical and radiographic parameters used. In the present study, P was defined according to the 2017 World Workshop [6], thus allowing a comparison of the obtained data with further studies to be carried out. Comparing the two groups of implant collar surfaces, a statistically significantly lower prevalence of P was found in the LMGS group. A previous study, at the 5-year follow-up examination, reported that implants with and without LMGS presented 3.6% and 11.9% of P, respectively (*p* < 0.05) [21]. It is well-documented in the literature that there is a negative impact of functional time on the onset of P [33,34]. Therefore, it is possible to suppose that the higher incidence of P recorded in the present study may be linked to a longer follow-up period (≥20 years).

Currently, available studies investigating the link between microbial implant contamination and the onset of peri-implant diseases indicated that the presence of bacterial insult alone is not sufficient for the development of P since the pathophysiology of P is strictly dependent on the subsequent subject’s immune response [6,35]. The conversion of PIM into P leads to an inflammatory extension through the peri-implant mucosa with the involvement of the implant-supporting bone. This often happens quickly and progressively because the peri-implant tissues present less functionally organized structures, resulting in reduced defense mechanisms, compared to periodontal tissues (lack of the periodontal ligament with the presence of only a narrow strip of circular fibers, which when exposed to bacterial attack causes loosening of the sealing) [36]. Some authors have hypothesized that a more functional organization of the peri-implant supra-crestal connective tissues could favor greater resistance of the same to the onset and progression of inflammation, preventing the evolution of PIM into P [14]. A logical interpretation of the present study’s findings could relate to the ability of the LMGS to ease a more functionally organized structure of the supra-crestal connective tissue during post-surgical healing. It is known that implants with a machined/smooth collar present a peri-implant scar-like tissue organization, with a circumferential arrangement of the connective fibers [36]. In reverse, histologic research in humans [18] documented around the implant with LMGS a different structural and functional organization of the supra-crestal connective tissues, with the presence of connective fibers perpendicularly oriented, which present a sort of physical attack into titanium laser-microtextured surface. The histologic features of ligature-induced lesions around implant collars with LMGS and without LMGS (machined) were examined in an experimental animal study by Rodrigues et al. [37]. The authors observed attachment of connective tissue fibers with a perpendicular orientation to LMGS, limiting the lesion extension and progression that protected the alveolar bone, whereas no connective fibers limited the lesion around the machined collar surface, which exhibited also a higher inflammatory infiltrate. The histologic organization and the physiologic functions of the supra-crestal peri-implant connective tissue play a fundamental role in the response to microbiological implant contamination, counteracting inflammatory progression [38,39]. Therefore, the histologic organization influences mechanical and biological defense mechanisms. The connective tissue attachment creates a physical barrier; consequentially, in its absence or poor functional organization (scar-like tissue), the conditions arise for easier apical migration of the inflammation. The LMGS promotes the formation of a sort of physical-mechanical seal, with fibers perpendicularly anchored to the surface of the implant collar, which promotes peri-implant soft tissue stabilization, which might, in turn, counteract the peri-implant inflammatory progression [37].

The influence of implant design (one-piece vs. two-piece) is being more often discussed as a possible risk factor of P. Some studies reported that one-piece implants have the capacity to maintain more stable hard and soft tissues around implants [38,39]. This could be linked to the presence of the micro-gap (usually located at the level of the bone crest) between implant and abutment in two-piece dental implants and its inevitable bacterial contamination, which could determine the onset of peri-implant tissue inflammation and P. It has been reported that healthy two-piece dental implants usually have deeper and wider peri-implant crevicular sulcus than one-piece dental implants, with a more active production of peri-implant crevicul fluid (PICF) [40,41]. Furthermore, the PICF of healthy one-piece dental implants is characterized by a lower pH than that of healthy two-piece implants [42]. These factors would determine the easiest development of peri-implant pathogenic bacteria, which grow more easily in slightly acidic environments [43,44]. Nevertheless, a recent meta-analysis [45] reported that two-piece implants have no significantly higher risk of P compared to one-piece implants, underlining that long-term randomized studies are still necessary to draw conclusions. 

According to the collected data, the lack of regular peri-implant maintenance, previously treated periodontitis, and smoking habits represent significant risk factors for the development of P. This is in accordance with data reported by several studies and systematic reviews [14,19,26,46,47] and suggests that these factors should be carefully considered when trying to prevent P. 

In the present study, cement-retained implant restorations compared to screw-retained restorations presented a higher prevalence of P, although the difference was not statistically significant. Reported data of a recent systematic review indicated that 33–100% of cement-retained implant restorations with P had excesses cement [48], which increases in volume and quantity the more the convexity of the emergence profile increases and the closer the prosthetic coronal margin gets to the implant platform [41,49]. Since cement residues are not only plaque-retentive but can also act as a foreign body [50], great care must be taken when cementing prostheses on implants [51,52]. 

Contradictory outcomes are reported in the literature on the relationship between hyperglycemia and P [53,54]. This could be justified by an imprecise distinction between the state of the disease (compensated and non-compensated) or by the lack of knowledge of the glycemic values reported by the patients. In the present study, no significant differences were found in the prevalence of P in diabetic vs. non-diabetic patients. However, the small number of diabetic patients in our sample may have influenced the significance of the result.

Contradictory data are present in the literature about the influence of tissue phenotype on the onset of P [14,55,56,57]. In the current study, the absence of a band of keratinized mucosa >2 mm was found to be a risk factor/indicator for P, but the difference was not statistically significant.

It is necessary to underline some limitations of the present study:

(1) the sample coming from a single private clinic.
(2) its retrospective design.
(3) the presence of several variables (some even reported in small numbers).

Consequentially, it could be assumed that the present study with the limitations mentioned above reports lower results with more variation than a study with a small, well-controlled, and selective group (efficacy study). However, studies like the present (effectiveness studies) may be more reflective of what can be expected from “routine practice”.

## 5. Conclusions

Within the limitations of this study, smoking habits, history of treated periodontitis, and lack of peri-implant maintenance were significant risk factors for P. Implants with a laser-microgrooved collar surface, compared with implants without a laser-microgrooved collar, presented a statistically significantly lower incidence of peri-implantitis.

## Figures and Tables

**Table 1:** Characteristics of patients diagnosed with PIM.

Systemic and Patient-Related Factors	N (%) Diagnosed PIM	Relative Risk (95% CI)	*p* Value *
Gender			
Male	223 (54.1)	1.28 (0.91, 1.73)	0.102
Female	189 (45.9)		
Presence of diabetes mellitus			
Yes	24 (5.8)	0.81 (0.51, 1.22)	0.415
No	388 (89.4)		
Presence of dyslipidemia			
Yes	36 (8.6)	0.88 (0.50, 1.53)	0.711
No	376 (91.4)		
Presence of hypertension			
Yes	28 (6.7)	1.05 (0.64, 1.72)	0.983
No	384 (93.3)		
Presence of osteoporosis			
Yes	4 (0.9)	2.91 (0.48, 16.84)	0.124
No	408 (93.3)		
Presence of anemia			
Yes	11 (2.6)	0.86 (0.33, 2.4)	0.772
No	401 (97.4)		
Presence of hypothyroidism			
Yes	9 (2.1)	1.16 (0.58, 2.72)	0.953
No	403 (97.9)		
Smoking habits			
Smokers	43 (10.4)	1.03 (0.63, 1.70)	0.984
Non-smokers	369 (89.6)		
History of treated periodontitis			
Yes	86 (20.8)	0.87 (0.61, 1.26)	0.456
No	336 (79.2)		
Lack of regular peri-implant maintenance			
Yes	289 (70.1)	3.14 (1.11, 42.4.3)	0.01
No	123 (29.9)		

CI: confidence interval. * Exact chi-square test.

**Table 2:** Characteristics of implants diagnosed with PIM.

Systemic and Patient-Related Factors	N (%) Diagnosed PIM	Relative Risk (95% CI)	*p* Value *
Collar			
LMCS	164 (45.6)	0.95 (0.71, 1.27)	0.622
no LMCSF	195 (54.4)		
Implant height (mm)			
<12	221 (61.5)	0.97 (0.72, 1.30)	0.803
≥12	138 (38.5)		
Implant diameter (mm)			
<4.2	187 (52)	0.95 (0.71, 1.27)	0.722
≥4.2	172 (48)		
Implant location			
Anterior maxilla	68 (18.9)	NA	0.318
Posterior maxilla	109 (30.3)		
Anterior mandible	50 (13.9)		
Posterior mandible	132 (36.7)		
Implant placement protocol [24,25]			
Type I	13 (18.9)	NA	0.48
Type II	20 (30.3)		
Type III	30 (13.9)		
Type IV	296 (36.73)		
Bone augmentation procedure at the time of implant placement			
Yes	108 (30.1)	1.12 (0.76, 1.65)	0.552
No	251 (69.9)		
Mucosal thickness and amount of attached keratinized mucosa (mm)			
≥2	148 (41.2)	1.62 (0.76, 2.05)	0.671
<2	203 (58.8)		
Type of prosthesis			
Single implant crown	179 (63.5)	1.26 (0.95, 1.68)	0.153
Multiple-unit implant-prosthesis	180 (33.5)		
Superstructure retention			
Screw-retained	168 (20.8)	0.93 (0.7, 1.25)	0.534
Cement-retained	191 (79.2		
Number of functional years prior to diagnosis			
<5 years	172 (47.9)	0.87 (0.61, 1.26)	0.456
5–10 years	178 (49.5)		
10–15 years	181 (50.4)		
≥15 years	187 (52.1)		
Lack of regular peri-implant maintenance			
Yes	310 (86.3)	6.14 (1.12, 64.33)	0.003
No	49 (13.7)		
Prosthetic complications			
Screw loosening	23 (6.4)	NA	0.414
Crown chipping	19 (5.2)		
Crown debonding	33 (9.1)		

CI: confidence interval. * Exact chi-square test.

**Table 3:** Characteristics of patients diagnosed with P.

Systemic and Patient-Related Factors	N (%) Diagnosed P	Relative Risk (95% CI)	*p* Value *
Gender			
Male	63 (45)	0.43 (0.09, 1.99)	0.312
Female	77 (55)		
Presence of diabetes mellitus			
Yes	19 (13.5)	0.76 (0.17, 3.49)	0.915
No	121 (86.5)		
Presence of dyslipidemia			
Yes	16 (11.4)	0.78 (0.31, 1.82)	0.911
No	124 (88.6)		
Presence of hypertension			
Yes	21 (15)	1.32 (0.17, 10.09)	0.798
No	119 (85)		
Presence of osteoporosis			
Yes	5 (3.5)	0.36 (0.05, 2.50)	0.924
No	135 (96.5)		
Presence of anemia			
Yes	6 (7.1)	0.20 (0.03, 1.26)	0.878
No	134 (92.9)		
Presence of hypothyroidism			
Yes	4 (2.8)	0.42 (0.06, 2.93)	0.222
No	136 (97.2)		
Smoking habits			
Smokers	96 (68.5)	9.24 (2.26, 37.60)	0.003
Non-smokers	44 (89.6)		
History of treated periodontitis			
Yes	94 (67.1)	7.31 (1.66, 29.47)	0.008
No	46 (32.9)		
Lack of regular peri-implant maintenance			
Yes	112 (80)	5.51 (1.12, 64.33)	0.002
No	38 (20)		

CI: confidence interval. * Exact chi-square test.

**Table 4:** Characteristics of implants diagnosed with P.

Systemic and Patient-Related Factors	N (%) Diagnosed P	Relative Risk (95% CI)	*p* Value *
Collar			
LMCS	13 (10.3)	8.36 (1.41, 92.14)	0.002
no LMCSF	113 (89.7)		
Implant height (mm)			
<12	60 (47.6)	1.62 (0.54, 4.91)	0.667
≥12	66 (52.4)		
Implant diameter (mm)			
<4.2	74(58.7)	1.39 (0.46, 4.20)	0.722
≥4.2	53 (41.3)		
Implant location			
Anterior maxilla	20 (15.8)		
Posterior maxilla	44 (34.9)	NA	0.07
Anterior mandible	20 (15.8)		
Posterior mandible	42 (33.4)		
Implant placement protocol [26]			
Type I	3 (2.4)		
Type II	7 (5.5)	NA	0.806
Type III	20 (15.8)		
Type IV	96 (76.1)		
Bone augmentation procedure at the time of implant placement			
Yes	56(44.4)	1.27 (0.29, 5.65)	0.752
No	70 (55.6)		
Mucosal thickness and amount of attached keratinized mucosa (mm)			
≥2	54 (42.8)	5.18 (0.76, 89.05)	0.451
<2	72 (57.2)		
Type of prosthesis			
Single implant crown	58 (46)	0.79 (0.22, 2.85	0.953
Multiple-unit implant- prosthesis	68 (54)		
Superstructure retention			
Screw-retained	49 (38.8)	2.39 (0.74, 7.78)	0.154
Cement-retained	77 (61.2)		
Number of functional years prior to diagnosis			
<5 years	47 (32.8)	0.87 (0.61, 1.26)	
5–10 years	52 (36.3)		0.456
10–15 years	66 (46.1)		
≥15 years	79 (62.9)		
Lack of regular peri-implant maintenance			
Yes	98 (77.7)	6.15 (1.21, 72.12)	0.002
No	28 (22.3)		
Prosthetic complications			
Screw loosening	20 (15.8)		
Crown chipping	24 (19)	NA	0.762
Crown debonding	31 (24.6)		

CI: confidence interval. * Exact chi-square test.

**Table 5:** Results of logistic regression analysis. B Coefficient (SE).

Predictor Variable	B Coefficient (SE)	Peri-Implantitis	Odds Ratio (95% CI)
		*p* Value	
no LMCS (yes 0, no 1)	2.61	0.005	6.42 (1.86, 51.30)
Smoking habits (yes 0, no 1)	2.34	0.007	9.14 (1.86, 51.30)
History of treated periodontitis (yes 0, no 1)	1.98	0.014	7.14 (1.48, 35.52)
Lack of regular peri-implant maintenance (yes 0, no 1)	2.44	0.026	11.33 (1.15, 93.69)

## Data Availability

The raw data are available upon request from Dr. Guarnieri or Dr. Reda.
